# Transport of the Dicyanogold(I) Anion

**DOI:** 10.1155/MBD.1994.433

**Published:** 1994

**Authors:** Katherine Tepperman, Yafei Zhang, Pamela W. Roy, Roger Floyd, Zheng Zhao, John G. Dorsey, R. C. Elder

**Affiliations:** 1 Department of Biological Sciences and Chemistry Laboratory of Clinical Virology and Biomedical Chemistry Research Center University of Cincinnati Cincinnati OH 45221-0006 USA

## Abstract

We have shown that dicyanogold(I), [Au(CN)_2_]^-^ is a common metabolite found in blood and urine samples of patients treated with different gold based drugs. Some patients have high levels of gold within their red blood cells (RBCs).   Size exclusion  and  C18  reversed phase chromatography show that the majority of the gold in RBC lysates is bound to protein, but small molecules such as dicyanogold(I) and gold-glutathione complexes are also present. Dicyanogold incubation with red blood cells in vitro leads to a rapid and complete uptake of gold. This uptake is unaffected by DIDS, an inhibitor of the anion channel, but is blocked by the addition of external cyanide. Dicyanogold is also readily taken up by H9 cells, a continuous CD^4+^ cell line. This uptake is significantly inhibited by N-ethylmaleimide, suggesting a requirement for sulfhydryl groups. Dicyanogold inhibits the replication of the AIDS virus, HIV, in a cell culture model.


### TRANSPORT OF THE DICYANOGOLD(I) ANION

Katherine Tepperman, Yafei Zhang, Pamela W.Roy, Roger Floyd, Zheng Zhao, John G. Dorsey and R.C. Elder

Department of Biological Sciences and Chemistry, Laboratory of Clinical Virology and Biomedical Chemistry Research Center, University of Cincinnati, Cincinnati, OH 45221-0006, USA

ABSTRACT

We have shown that dicyanogold(I), $[Au(CN)_2]^-$ is a common metabolite found in blood and urine samples of patients treated with different gold based drugs. Some patients have high levels of gold within their red blood cells (RBCs). Size exclusion and C18 reversed phase chromatography show that the majority of the gold in RBC lysates is bound to protein, but small molecules such as dicyanogold(I) and gold-glutathione complexes are also present. Dicyanogold incubation with red blood cells in vitro leads to a rapid and complete uptake of gold. This uptake is unaffected by DIDS, an inhibitor of the anion channel, but is blocked by the addition of external cyanide. Dicyanogold is also readily taken up by H9 cells, a continuous $CD4^+$ cell line. This uptake is significantly inhibited by N-ethylmaleimide, suggesting a requirement for sulfhydryl groups. Dicyanogold inhibits the replication of the AIDS virus, HIV, in a cell culture model.

INTRODUCTION

Gold-based drugs have been used for over sixty years for the treatment of rheumatoid arthritis. They are among only a few drugs which have been tested in a double blind study and shown to be effective in inducing remission of the disease (1). In spite of the reported efficacy of the drugs, there is no clear understanding of their mechanism of action. It is also unclear why some individuals experience severe toxicity under the same regimen which gives others positive results. For several years, we have been interested in developing methods to detect gold-containing metabolites in patient fluids. Based on an in vitro experiment to monitor intestinal uptake of auranofin (triethylphosphinegold(I)tetraacetylthioglucose, AF), we showed that it was a metabolite of the drug and not the drug itself which crosses the intestinal wall (2). More recently, we have developed methods using high performance liquid chromatography, HPLC, interfaced to an inductively coupled plasma-mass spectrometer, ICP-MS, for gold-specific detection to analyze blood and urine samples from patients (3-5). These experiments have shown that the drugs themselves do not circulate in patients to any significant extent. Thus, gold effects are due to gold-containing metabolites.

Recently, we have identified one of the gold-containing metabolites found in patient fluids (6), dicyanogold(I), $[Au(CN)_2]^-$. This compound was found first in urine samples from a patient being treated with myochrysine (sodium gold thiomalate, AuTm) but was subsequently found in both blood and urine samples from patients being treated with both solganol (gold thioglucose, AuTg) and AF. This represented the first identification of gold-containing metabolites in patient fluids and the first evidence that the three different gold-based drugs have common metabolites. Dicyanogold was found at the highest levels in patients who reported that they smoked, but was also found in reported nonsmokers.

Patients treated with myochrysine show wide variation in the amount of gold found in their red blood cells (RBCs)(7). Graham, et al. (8) correlated the high levels of gold in RBCs with patient smoking. They also showed that cyanide increases the amount of gold taken up by RBCs incubated with AuTm in vitro. Thus, they suggested that the increase in gold uptake by red cells in smoking patients was due to the formation of dicyanogold as a result of reaction of cyanide in the blood with gold complexes.

Since we have now demonstrated that dicyanogold(I) is found in the bloodstream of patients (6) and that the presence of this species in blood seems more prevalent among smokers, we have begun to study in more detail the interaction of dicyanogold(I) with cells. This report describes experiments with RBCs which show rapid and complete uptake of gold after in vitro incubation with dicyanogold(I). The fact that gold is readily taken up by RBCs suggests that it may also be taken up by other cells. Of particular interest are T-lymphocytes, which serve as the site of replication of the AIDS virus, HIV. Previous reports had suggested that the reverse transcriptase of HIV can be inhibited by gold complexes such as AuTg. However, this compound cannot enter cells readily and therefore cannot reach the intracellular concentrations necessary to inhibit the enzyme. The uptake of gold from dicyanogold(I) incubation could result in internal gold concentrations sufficient to inhibit HIV replication. We have used a cell culture model system to explore this possibility, using H9 cells, a $CD4^+$ established cell line which can support the replication of HIV. We have monitored the effects of dicyanogold(I) on these cells and show preliminary results which indicate that incubation of H9 cells with dicyanogold(I) can inhibit the replication of HIV.

### MATERIALS AND METHODS

*Chemicals:* Cell culture reagents were purchased from GIBCO (Grand Island, N.Y.). Sodium gold(I) thiomalate (AuTm), 4,4'-diisothiocyanatostilbene-2, D,L-sulfonic acid (DIDS), and N-ethylmaleimide (NEM) were purchased from Aldrich Chemicals (Milwaukee, WI). Auranofin (AF) was provided by SmithKline Beecham (Philadelphia, PA). Potassium dicyanogold(I) ($KAu(CN)_2$) and tetrabutylammonium chloride (TBAC) were purchased from Sigma Chemical Co. (St. Louis, MO). A standard protein mixture was obtained from BioRad Laboratories (Hercules, CA). Sodium dodecyl sulfate (SDS) was purchased from Fluka Biochemika (Switzerland). All chemicals used for mobile phases were purchased from commercial sources and were of analytical grade. All water for chromatography was purified (18 MΩ resistance reading) through a Barnstead Nanopure system (Milford, MA) equipped with a 2 μM filter.

*Instrumentation*: The HPLC system used included a Spectra-Physics 8800 ternary HPLC pump, a Rheodyne 7125 injector with a 10 or 100 μL sample loop, an Applied Biosystems 757 variable wavelength detector and a Houston Instruments (Austin, TX) strip chart recorder. Gold specific detection utilized a Sciex Elan 250 inductively coupled plasma-mass spectrometer (ICP-MS). The RF power was 1.4 kW, nebulizer Ar flow was 1 L $min^{-1}$, and the nebulizer spray chamber was cooled at -10° C to condense most of the organic vapor. Gold was monitored at 197 M/Z. For some experiments, FIA was done using a Fisons VG Plasma Quad II, with RF power at 1.35 KW, nebulizer air at 1 L/min or a Fisons VG Plasma Quad I, RF power at 1.7 KW, nebulizer air at 1 L/min. The column was connected to the nebulizer by PTFE tubing (100 cM x 0.1 mM i.d.). Data were collected in the multielement mode with an IBM PS/2 computer.

*Chromatography and Flow Injection Analysis*: An analytical C18 column (Adsorboshpere C18, Alltech), 5μm, 300 x 4.1 mm was used to analyze the low molecular weight materials. The mobile phase consisted of 1:1 methanol-water, 5 mM phosphate buffer (pH 7.3) and 10 mM TBAC as an ion pairing agent. Prior to use, the mobile phase was filtered through a 0.45 μM Nylon-66 membrane filter. The column was equilibrated with the mobile phase for at least 1 hour. All separations were performed at a flow rate of 1 mL $min^{-1}$ at room temperature in an isocratic mode. High molecular weight materials were analyzed using a size exclusion column, Biosil 250-5 (300 x 78 mm, nominal molecular weight range: 10,000-300,000 Da). The mobile phase contained 0.05 M $Na_2SO_4$ or NaCl, 0.01 M phosphate buffer, pH 7.3, and 0.05% $NaN_3$. Flow injection analysis was done by connecting a Rheodyne injector with a 10 μL loop to the ICP-MS using PTFE tubing. Ion counts were compared to standards ranging in concentration from 10-600 ppb.

*Patient Samples:* Clinical blood samples and urine samples were obtained from rheumatoid arthritis patients being treated with myochrysine, auranofin or solganol at the Arthritis Clinic, University of Cincinnati Medical Center under a protocol approved by the Institutional Review Board of the University. Samples were taken as part of the routine examinations of the patients. For those receiving myochrysine or solganol, the blood samples were drawn prior to administration of the weekly injection. For the experiments involving in vitro incubation of blood cells, blood was obtained from a healthy volunteer. All blood samples had EDTA as an anticoagulant. Plasma was frozen, and red blood cells were kept at 4° C until use. For C18 analyses of low molecular weight materials, serum or lysate solutions were centrifiltered at 4000 rpm in an IEC Centra-4B centrifuge for two hours, using a filter with a molecular weight cut off of 10,000 Da (Alltech). Urine samples were filtered through a 0.45 $\mu$ filter as soon as possible after collection, then stored at 4°C until analysis.

*Red Blood Cell Incubations*: Experiments were performed using either whole blood or washed red blood cells (RBCs). Washed RBCs were prepared by centrifuging whole blood at 1500 g for 5 min, then resuspending the cells in phosphate buffered saline, PBS (0.8% NaCl, 0.02% $KH_2PO_4$, and 0.116% $Na_2PO_4$, w/v). Cells were then centrifuged, and the PBS wash was repeated five times. The cells were finally resuspended in PBS at a final volume equal to the volume of blood from which the cells were taken. Incubations with gold compounds were carried out by adding the compound to either whole blood or washed RBCs, then incubating at 37° C for 25 min. The incubation was stopped by centrifugation of the samples, separating the cells from the supernatant (either plasma fraction or PBS) for subsequent analyses. Cell lysates were prepared after washing the cells five times in PBS, by addition of distilled water to the cell pellet or by ultrasound. Lysates were separated from the membrane fraction by centrifugation at 15,000 g for 30 min.

*H9 cell cultures*: H9 cells were obtained from the American Type Culture Collection (Rockville, MD). Cells were maintained in "complete medium" containing RPMI 1640 and 20% newborn calf serum at 37° C in a NAPCO water jacketed incubator with an atmosphere of 5% $CO_2$ and 95% air. Cells were counted using a hemocytometer. Medium was regularly replenished and the cells were maintained between $10^5$ and $10^6$ cells per mL. For incubations with dicyanogold(I), cultures were established at $10^5$ cells per mL, then dicyanogold(I) was added to the indicated concentrations. At daily intervals, cells were counted using the hemocytometer. Cell viability was determined by trypan blue dye exclusion.

*HIV culture, reverse transcriptase assays*: All work was carried out in the P3 facility of the Laboratory of Clinical Virology. Cells from a logarithmically growing H9 culture were centrifuged at 800 RPM for 10 min, then counted and resuspended at 1 x $10^6$ cells per mL. 2 mL was added to each of 2 centrifuge tubes and cells were pelleted as above. Cell pellets were resuspended in 0.4-0.5 mL of HIV inoculum and incubated at 37° C for two hours, resuspending the cells gently at 30 min intervals. Cells were then pelleted, then washed, pelleted again, and suspended in 10 mL of fresh medium (2 x $10^5$ cells/mL). 20 ppb dicyanogold(I) was added to one culture. Cultures were placed in 25 $cm^2$ flasks and incubated at 37° C with a 5% $CO_2$ atmosphere. At 3-4 day intervals, cells were counted, assessed for viability, and diluted to 2 x $10^5$ cells per mL. At each time point, 1 mL of growth medium was saved after removing the cells by centrifugation. These medium samples were saved and stored for assays of reverse transcriptase activity (10).

### RESULTS AND DISCUSSION

We have used FIA with ICP-MS detection to monitor gold levels in plasma and urine samples from patients treated with gold-based antiarthritis drugs. Most of our work has been with samples from patients on AuTm therapy, since that is the principal gold drug administered at the Arthritis Clinic at the University of Cincinnati. Gold levels in AuTm patients show a steady increase from the beginning of therapy until reaching a steady state level at about 30 days (following weekly injections). Gold levels in plasma samples are typically around 2 ppm, once the steady state level has been reached. Although there seems to be relatively little variability among patients in plasma gold levels, gold levels in the RBC lysates are extremely variable. In two patients at the same stage of treatment with the same treatment regimen, one has gold levels in the RBC lysates that are 15% of the level in the plasma, while another has levels essentially identical to those in the plasma. These differences may not be directly attributable to smoking, since both of these patients report that they are occasional smokers. However, independent verification of smoking frequency may be required to demonstrate a connection.

Red blood cell lysates from patients have been examined by HPLC to determine the intracellular binding sites for the gold. Figure 1 shows a size exclusion chromatogram of a RBC lysate. The upper trace shows the protein peaks identifiable by UV detection at 254 nm. The major peak has a retention time commensurate with hemoglobin, based on protein standards. Gold-specific detection by ICP-MS (lower trace) shows one major gold-containing peak, as well as some poorly resolved peaks at lower molecular weight. Some of the gold elutes at the retention time expected for hemoglobin, but the major peak elutes earlier, at about 7 min. Based on

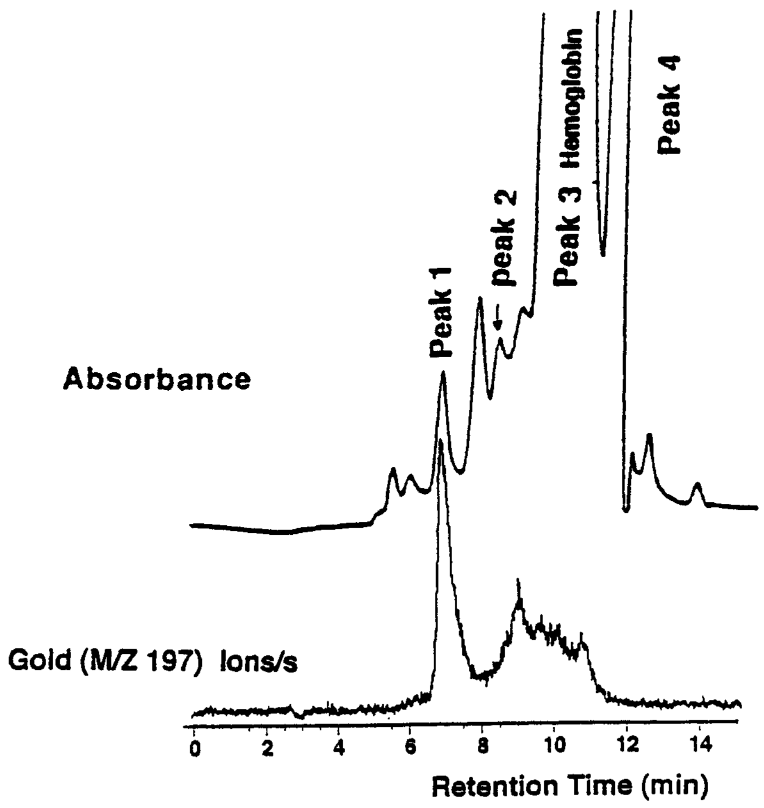

Figure 1 Size exclusion separation of patient red blood cell lysates. Lysate was separated on a Biosyl 250-5 column. Upper trace, UV detection at 254 nM, lower trace, gold-specific detection with the ICP-MS.

comparison to standards, this material has a molecular weight of approximately 330,000 Da. The gold peak corresponds to a relatively minor protein peak as judged by its small absorbance. Since hemoglobin is the major protein present in the red cell, these higher molecular weight materials must bind gold much more efficiently than hemoglobin.

Low molecular weight gold-containing species in the patient red blood cell lysates were separated using C18 reversed phase chromatography. Before injection onto the column, the lysates were filtered through a membrane with a molecular weight cut off of 10,000 Da. Approximately 3% of the total gold in the lysate was found in the low molecular weight fraction. Figure 2 shows a separation of a RBC lysate solution. There is a significant gold peak which is retained very little or not at all by the column, probably consisting of ionic or polar molecules. In addition at least four peaks can be seen. The peak which elutes at 10 minutes has the retention time of a dicyanogold(I) standard. Two of the additional peaks correspond to gold species found when either AuTm (upper trace) or $Au(CN)_2$ (middle trace) is reacted with glutathione. Since glutathione levels in red blood cells are relatively high (2-3 mM), it seems likely that gold glutathione complexes would be present. In summary, the lysate from red blood cells of a patient

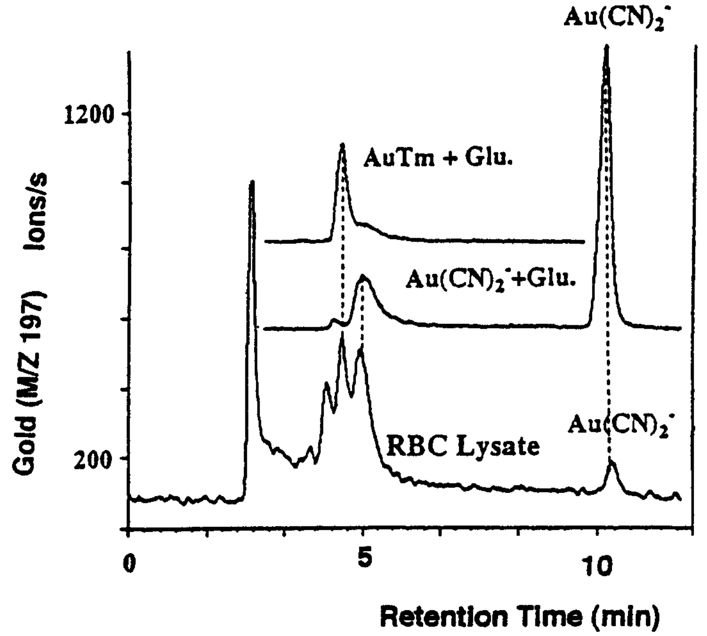

Figure 2 C18 chromatography of patient red blood cell lysates. The lysate was centrifiltered to isolate low molecular weight materials, then separated on a C18 column with a mobile phase of 1:1 methanol-water, 5 mM phosphate buffer and TBAC as an ion pairing agent. Gold was detected with the ICP-MS.

being treated with myochrysine contains high levels of gold. The majority of the gold is bound to high molecular weight materials, a minor constituent has the retention time for hemoglobin. Another of the proteins has an apparent molecular weight of 300,000 Da. Among the low molecular weight gold species present, there are gold peaks which correspond to dicyanogold(I) and also to a bis(glutathione)gold(I) complex.

Table 1. Incubations with dicyanogold. Whole blood or RBCs were incubated for 25 min at 37°C. Fractions were analyzed by FIA with ICP-MS detection.

| | Whole blood (% total gold) | Washed Cells (% total gold) |
|---|---|---|
| Supernatant | 17 | 1 |
| Lysate | 78 | 92 |
| Membranes | 5 | 6 |

Based on the presence of dicyanogold(I) in patient blood and on previous work suggesting that cyanide facilitates the uptake of gold, we have examined gold uptake by RBCs after in vitro incubation with dicyanogold(I). Whole blood or washed red blood cells were incubated with 130 ppb dicyanogold(I) at 37° C for 25 min. At the end of the incubation, fractions were prepared as described above, and analyzed for gold by FIA using the ICP-MS. Table 1 shows the results of these incubations. With either whole blood or washed cells, most of the gold in the system was found in the lysates. The whole blood samples had significantly more gold in the supernatant after the incubation, probably reflecting protein binding. This rapid and complete uptake of gold after dicyanogold(I) incubation is in contrast to similar experiments in which the cells were incubated with AuTm. In that case, red cell lysates from incubation with either whole blood or washed RBCs had taken up about 5% of the total gold in the medium.

The mechanism of gold uptake from dicyanogold is not understood. One possibility is that dicyanogold, a small anion, could enter the red blood cells via the anion channel in the membrane. We tested this possibility by using an irreversible inhibitor of the red blood cell anion channel, 4,4'-diisothiocyanatostilbene-2,2'-sulfonic acid (DIDS) (9). Table 2. shows the results of incubation of washed red blood cells with 70 μM DIDS.

**Table 2.** Effect of DIDS and NaCN on uptake of dicyanogold by RBCs. Washed RBCs were incubated with 900 ppb dicyanogold at 30° C, following preincubation for 20 min in PBS alone or with 70 μM DIDS or 0.67 mM NaCN. Fractions were analyzed by FIA and ICP-MS. Standard deviations are listed for each value.

| Inhibitors | PBS (% total gold) | DIDS (% total gold) | NaCN (% total gold) |
|---|---|---|---|
| Supernatant | 1.8 ± 1.3 | 0.8 ± 0.1 | 81.3 ± 0.3 |
| Lysate | 94.4 ± 1.8 | 94.4 ± 0.9 | 18.4 ± 0.4 |
| Membranes | 4.7 ± 1.6 | 4.9 ± 1.0 | 0.4 ± 0.2 |

Preincubation of the RBCs with DIDS had no effect on uptake of gold by the cells. This shows that dicyanogold does not enter the red blood cells through the anion channel.

Table 2 also shows the results of another experiment in which the effects of added $(CN)^-$ on gold uptake was tested. Preincubation of the RBCs with NaCN causes a significant inhibition of gold uptake on dicyanogold(I) incubation. In this case, most of the gold remains in the supernatant. The fact that external cyanide inhibits gold uptake in this experiment demonstrates that the mechanism of uptake involves the loss of cyanide from dicyanogold(I).

The observation that RBCs incubated with dicyanogold(I) show rapid and extensive uptake of gold suggested the possibility that dicyanogold might be useful in the treatment of AIDS. In vitro experiments on reverse transcriptase, the replicating enzyme of the AIDS virus, HIV, have shown that AuTg can inhibit the enzyme (11). AuTg, like AuTm. does not readily enter cells. Thus, the concentrations necessary to inhibit the reverse transcriptase enzyme in vitro are not achieved within cells. Since HIV replication takes place within cells, only a compound which provides effective uptake of gold can be expected to inhibit the replication of the AIDS virus. Dicyanogold(I) is a likely candidate, based on our results with red blood cells.

To explore the effects of dicyanogold on HIV replication, we began by studying the effects of the compound on a $CD4^+$ continuous cell line, H9 cells. This line was developed in Robert Gallo's laboratory (12). The line was selected because the cells permit the replication of strains of HIV. Initial experiments tested the effects of different concentrations of dicyanogold(I) on the growth rate of the H9 cells (Figure 3). Cells were cultured with dicyanogold concentrations

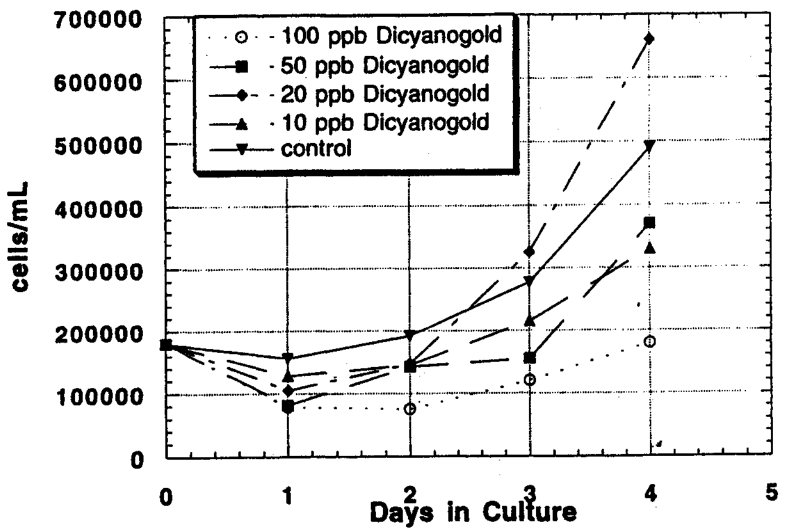

**Figure 3** Effects of Dicyanogold(I) on growth of H9 cells. Cells were incubated in varying concentrations of dicyanogold(I). At daily intervals, cell number was determined with a hemocytometer.

ranging from 10 to 100 ppb. After an initial lag, the cells with no added dicyanogold increased with an apparent doubling time of approximately 22 hours. Cells exposed to 10 ppb or 20 ppb dicyanogold(I) doubled at control rates. The cells grown in 50 ppb, showed a more significant lag before an increase in number was observed. However, by the third day of culture, doubling times approached control levels. The cells exposed to 100 ppb dicyanogold began to grow after several days, but did not grow at control rates during this experiment. In later experiments, cells were maintained in 100 ppb for longer periods and after an initial lag doubled at control rates.

We also examined the effects of dicyanogold(I) on H9 cells using the criterion of cell viability rather than growth rate. Cells were again exposed to varying concentrations of dicyanogold(I) and at regular intervals cells in each culture were tested for cell viability using the trypan blue dye exclusion method. Figure 4 shows that the initial effects on cell viability clearly correlate with the observed effects on growth rate. It should be noted that in this and subsequent figures, the lines drawn are to aid the eye and do not suggest the response of the intermediate points. Approximately 90% of the cells in the culture with 25 ppb dicyanogold(I) were viable on day three, essentially the control level. Cells in 50 ppb showed a slight decrease in viability initially, but control levels of viability were observed after three to five days. In contrast, 75 and 100 ppb dicyanogold(I) caused a significant decrease in cell viability. In these cases, however, cells rapidly recovered. Within one week, in cultures in 75 ppb Au 90% of the cells were viable, and in cultures with 100 ppb Au the value rose to 80%. This result indicates that the cells are able to tolerate high levels of dicyanogold(I). This observation is consistent with our observations of patient samples. We have detected levels of dicyanogold as high as 100 ppb in patient samples. These patients were exhibiting no apparent toxic effects, suggesting that dicyanogold is well tolerated by patients.

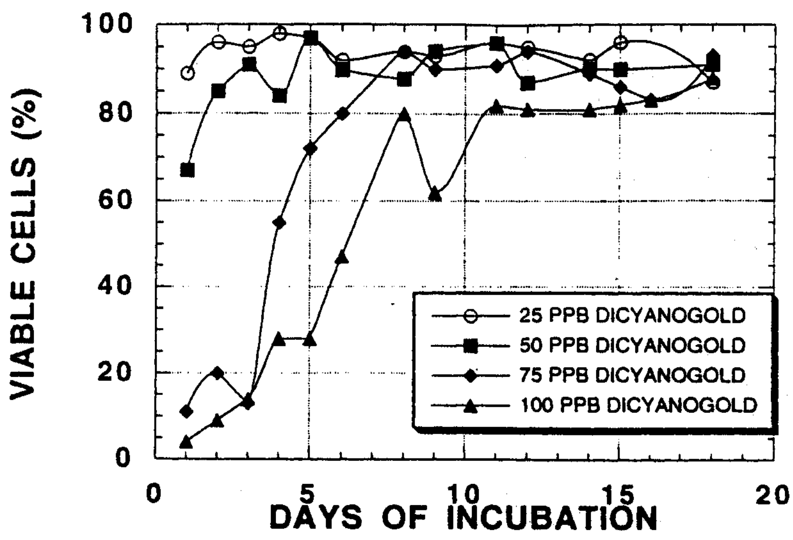

**Figure 4** Effect of dicyanogold(I) on H9 cell viability. Cells were incubated in varying concentrations of dicyanogold(I). At regular intervals, cell viability was assessed by trypan blue dye exclusion.

Since H9 cells can tolerate relatively high levels of dicyanogold(I), we were interested in determining the extent of uptake of gold by H9 cells exposed to this compound. A series of cultures was set up with 8.7 x $10^5$ cells per mL. These cultures were incubated with varying concentrations of dicyanogold(I) for one hour at 37°C. At the end of the incubation, cells were removed by centrifugation. Cell pellets were washed in PBS, then cells were lysed as was done with the red blood cells. Cell lysates were assayed for gold amounts by FIA with the ICP-MS. Figure 5 shows that the cells exposed to dicyanogold accumulated gold to levels dependent on the external concentration. In all cases, the amount of gold found within the cell lysates was at least 70 fold greater than the concentration in the corresponding incubation medium. *This shows that the H9 cells concentrate gold when incubated with dicyanogold. This is a critical result if the cells are to achieve gold concentrations necessary to inhibit HIV.*

H9 cells were also used to further investigate the mechanism of uptake of gold from dicyanogold(I) incubation. It has been proposed that gold compounds may enter cells via a "sulfhydryl shuttle" (13). In this model, gold complexes undergo ligand exchange to bind to sulfhydryl groups in or on the cell membrane. In a series of reactions, the gold passes through the membrane where it is then released into the cytosol. The sulfhydryl shuttle is expected to be inhibited by compounds which block surface sulfhydryl groups, such as N-ethylmaleimide (NEM). To test whether blocking surface sulfhydryl groups interferes with gold uptake in H9 cells, the cells were incubated with 1 mM NEM at 37° C for 30 min. The NEM was then removed by centrifugation of the cells. The cells were rinsed in complete medium, centrifuged again, then resuspended in complete medium and exposed to varying concentrations of dicyanogold(I) as above. As shown in Figure 5, NEM significantly blocks the uptake of gold in this experiment. This result is consistent with the sulfhydryl shuttle mechanism of uptake.

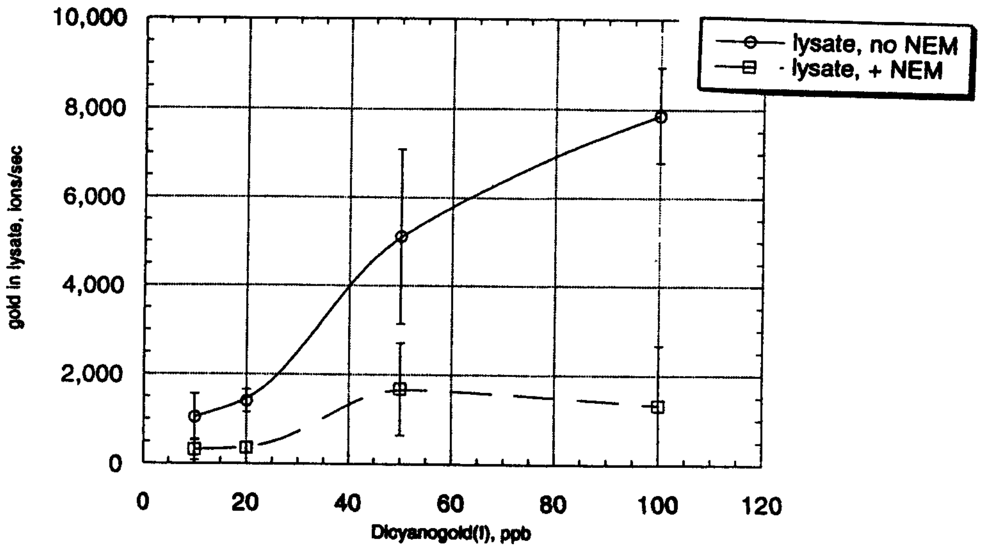

**Figure 5** Uptake of gold in H9 cells incubated with dicyanogold. Cells were incubated in control medium or medium containing 1mM NEM for 30 min. They were then incubated in varying concentrations of dicyanogold(I). Cell lysates were prepared and analyzed for gold by FIA-ICP-MS.

Since we have shown that H9 cells can tolerate relatively high levels of dicyanogold(I) and that the cells appear to concentrate gold when exposed to this compound, we then tested whether dicyanogold could inhibit the replication of HIV. H9 cells were exposed to HIV on day 0. The control culture contained only HIV, while the experimental one contained both HIV and 20 ppb dicyanogold(I). At regular intervals, cells were collected from the culture, lysed and assayed for reverse transcriptase activity as a measure of HIV development. Figure 6 shows the results. In cells with no gold, there is a significant increase in the amount of reverse transcriptase which peaks at about seven days after infection. The cells which were incubated in 20 ppb dicyanogold have a reverse transcriptase which is about 50% of the control level at seven days. *Clearly this preliminary result suggests that dicyanogold can, indeed, inhibit the replication of the AIDS virus.*

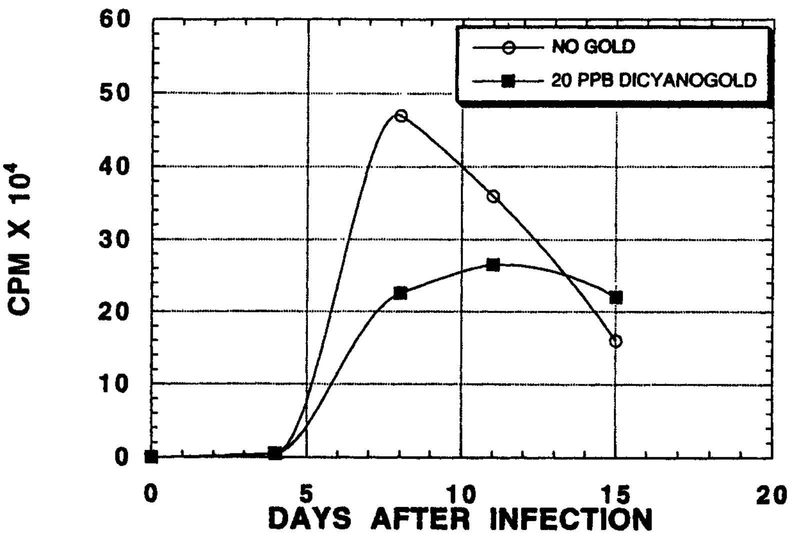

**Figure 6** Effects of dicyanogold on HIV replication. H9 cells were infected with HIV. One culture contained normal medium, one contained 20 ppb dicyanogold. At indicated intervals, cells were assayed for reverse transcriptase activity.

### CONCLUSIONS

We have used FIA with ICP-MS detection to monitor gold levels in patients being treated with gold-based antiarthritis drugs. Our studies show that gold levels in patient blood samples generally are in the range of 0.5-3 ppm. Although patients on myochrysine may have comparable levels of gold in their blood plasma, they vary in the amount of gold in their red blood cells. This observation is consistent with previous findings (7,8). By chromatographic analysis of plasma samples from these patients, we have shown that dicyanogold is found in the blood independent of the original drug administered. Graham, et al. (8) had predicted with dicyanogold was a likely metabolite, especially in smokers. While we see higher levels of dicyanogold in smokers, we can also detect it in non-smoking patients.

Analysis of red blood cell lysates from patients shows that the majority of the gold is bound in high molecular weight form. Although some of the gold appears to be bound to hemoglobin, the gold is also found in a higher molecular weight fraction. Among the low molecular weight gold species present in the red blood cell lysates are dicyanogold(I), and bis(glutathione)gold(I) complexes.

In vitro studies show that dicyanogold incubation results in rapid uptake of gold by red blood cells. This uptake is not affected by the inhibitor of the anion channel, DIDS, which shows that dicyanogold does not enter the cells through the anion channel. Excess cyanide does inhibit uptake of gold in dicyanogold incubations, which indicates that uptake involves the loss of a cyanide moiety.

Studies on the $CD4^+$ cell line, H9, confirm that incubation of cells with dicyanogold leads to significant uptake of gold. The uptake of gold is concentration dependent, and cell lysates have gold levels at least 70 fold higher than the external medium. This concentration of gold by cells is significantly inhibited by pretreatment of the cells with NEM. The fact that blocking surface sulfhydryls affects gold uptake is consistent with the proposed sulfhydryl shuttle mechanism (13).

The ability of H9 cells to accumulate gold as a result of incubation with dicyanogold(I) suggests that dicyanogold might be useful in the treatment if AIDS. H9 cells seem to tolerate relatively high levels of dicyanogold. *The fact that high levels are tolerated in patients as well makes dicyangold a good candidate for further testing.* Our preliminary studies indicate that even the relatively low level of dicyanogold(I) of 20 ppb can inhibit the replication of HIV in vitro. Further studies are clearly warranted to determine whether dicyanogold(I), either alone or in combination with other agents, will provide a useful therapeutic for AIDS.

ACKNOWLEDGEMENTS

We would like to thank Dr. Keith Pryhuber and Mary Nordlund for supplying the patient samples. The ICP-MS was purchased with a Research Challenge Grant from the State of Ohio. Zheng Zhao and Yafei Zhang thank the Department of Chemistry for graduate fellowships.

REFERENCES

1. Empire Rheumatism Council, *Ann. Rheum. Dis.* **20**, 315-33 (1961).
2. Tepperman, K., Finer, R., Donovan,S., Elder, R.C., Doi, J., Ratliff, D., Ng, K., *Science* **225**, 430-432 (1984).
3. Matz, S.G., Elder, R.C., Tepperman, K., *J. Anal. Atom. Spectrom.* **4**, 767-771 (1989).
4. Elder, R.C., Jones, W.B.,Tepperman, K., *A.C.S.Symp Ser.* **479**, 309-325 (1992).
5. Zhao, Z., Jones, W.B., Tepperman, K., Dorsey, J.G., Elder, R.C., *J. Pharm. Biomed. Anal.* **10**, 279-87 (1992).
6. Elder, R.C., Zhao, Z., Zhang, Y., Dorsey, J.G., Hess, E.V., Tepperman, K., *J. Rheumatol.* **20**, 268-2727.
7. Smith, P.M., Smith, E.M., Gotlieb, N.L., *J. Lab. Clin. Med.* **82**, 930-937 (1973)
8. Graham, G.G., Haavisto, T.M., Jones, H.M., Champion, G.D., *Biochem. Pharmacol.* **33**, 1257-1262 (1984).
9. Ship, S., Shami, Y., Beuer, W., Rothstein, A., *J. Memb. Biol.* **33**, 311-323 (1977).
10. Spira, T.J., Bozeban, L.H.,Holman, R.C., Warfield, D.T., Phillips, S.K., Feorine, P.M., *J. Clin. Microbiol.* **25**, 97-99.
11. Blough, H., Richetti, M., *International Patient*, W090/14825 (1990).
12. Gallo, R.C., Popovic, M., *Science* **224**, 497-500 (1984).
13. Snyder, R.M., Mirabelli, C.K., Crooke, S.T., *Biochem. Pharm.* **35**, 923-932 (1986).

